# The Evaluation of the 1318 nm Diode Laser in Open Liver Surgery

**DOI:** 10.3390/cancers14051191

**Published:** 2022-02-25

**Authors:** Patrick Pfitzmaier, Matthias Schwarzbach, Ulrich Ronellenfitsch

**Affiliations:** 1 Department of General, Visceral, Vascular and Thoracic Surgery, Klinikum Frankfurt Höchst, Gotenstraße 6-8, 65929 Frankfurt, Germany; patrick.pfitzmaier@klinikumfrankfurt.de (P.P.); matthias.schwarzbach@klinikumfrankfurt.de (M.S.); 2 Department of Visceral, Vascular and Endocrine Surgery, University Hospital Halle (Saale), Martin-Luther-University Halle-Wittenberg, 06120 Halle, Germany

**Keywords:** hepatectomy, hepatic resection, laser, morbidity, complications, mortality, blood loss, pringle, surgery

## Abstract

**Simple Summary:**

The surgical treatment of liver metastases but also of primary liver tumors is of particular clinical relevance in view of their high incidence. As a therapy option with a prospect of long-term survival in curative intent, liver resection plays a crucial role in modern multimodal treatment concepts. Advances in surgical and perioperative medicine have led to a reduction of procedural mortality to less than 5% and morbidity to around 35–45% and have rendered liver resections from high risk to routine procedures. Several comparisons of common parenchymal dissection techniques showed no specific advantages in favor of one of the methods in terms of morbidity, mortality and intraoperative blood loss. The aim of our retrospective study was to evaluate the 1318 nm diode pumped neodymium-yttrium aluminum garnet laser in open liver surgery. It could be shown that liver resections using the 1318 nm surgical laser can be carried out with an acceptable risk and are equally safe compared to conventional resection methods with comparatively little blood loss and favorable oncological surrogate outcomes.

**Abstract:**

(1) Background: Numerous dissection instruments are available for liver resection. So far, there has been no evidence in favor of a specific dissection device effecting a reduction in postoperative mortality and morbidity or a reduction in intraoperative blood loss. The aim of the study was to evaluate the safety of liver resection with the 1318 nm surgical laser. (2) Methods: 151 consecutive patients who underwent liver resection using the 1318 nm surgical laser (*n* = 119) or conventional dissection methods (*n* = 32) were evaluated retrospectively. As primary outcome, postoperative complications were assessed using the Clavien–Dindo classification. Secondary outcomes were postoperative mortality, reoperations and reinterventions, intraoperative blood loss, the need for vascular control using the Pringle maneuver and oncological safety assessed through histopathological evaluation of resection margins. (3) Results: For liver resections using the 1318 nm surgical laser, the postoperative morbidity (41.2% vs. 59.4%, *p* = 0.066), mortality (1.7% vs. 3.1%, *p* = 0.513) and the reoperation rate (2.5% vs. 3.1%, *p* = 1.000) were not significantly different from conventional liver resections. In the laser group, a lower reintervention rate (9.2% vs. 21.9%, *p* = 0.050) was observed. The oncological safety demonstrated by a tumor-free resection margin was similar after laser and conventional resection (93.2% vs. 89.3%, *p* = 0.256). The median intraoperative blood loss was significantly lower in the laser group (300 mL vs. 500 mL, *p* = 0.005) and there was a significantly lower need for a Pringle maneuver (3.4% vs. 15.6%, *p* = 0.021). (4) Conclusions: Liver resections using the 1318 nm surgical laser can be routinely performed with a favorable risk profile. Compared to alternative resection methods, they are associated with low blood loss, appear adequate from an oncological point of view, and are not associated with increased mortality and morbidity.

## 1. Introduction

The surgical treatment of liver metastases, for example, from colorectal cancer, as well as primary liver tumors, is of particular clinical relevance in view of their high incidence and the oncological benefit associated with resection in many settings. In addition to general operability and disease-specific factors such as histology and the presence of extrahepatic metastasis, the oncologically complete removal of malignant tumors (“R0 resectability”) plays a decisive role [1,2,3,4,5,6]. With an increasing number of early, resectable findings, liver resections have gained in frequency and importance in recent decades [6,7,8]. The prospect of improved outcome with long-term survival and sometimes cure makes liver resections an important part of modern, multimodal therapy concepts [9]. Advances in perioperative medicine, an indication based on validated criteria, the increase in surgical experience as well as the improvement of surgical technique have lowered perioperative mortality to <5% and morbidity to 35–45% and transformed the former high-risk procedure to an established standard [6,8,10,11,12,13,14,15]. Notwithstanding the improved procedural safety and perioperative treatment, liver resections remain challenging. A Cochrane meta-analysis from 2009 comparing common dissection devices could not prove any advantage in favor of a specific device regarding postoperative mortality and morbidity or intraoperative blood loss [9,16,17]. The use of the 1318 nm laser (Eraser 1318 nm, Rolle + Rolle GmbH & Co KG, Salzburg, Austria) as a dissection instrument has been established in lung surgery. Studies have shown improved long-term results with low complications [18,19]. The routine clinical application of the 1318 nm laser in liver surgery has not yet been adequately evaluated. The aim of this retrospective study was to evaluate the use of the 1318 nm surgical laser compared to conventional resection in open liver surgery.

## 2. Materials and Methods

### 2.1. Ethics Approval

Ethics board approval was obtained from the Medical Ethics Commission II of the Medical Faculty Mannheim, Heidelberg University, Mannheim, Germany (2016–855R-MA, approval date: 18 October 2016). All patient data used in this analysis were completely anonymized. The study was performed according to the Declaration of Helsinki.

### 2.2. Study Design

The present study is a single-center retrospective cohort study using data from a prospectively kept institutional database.

### 2.3. Patients

**Figure 1:**
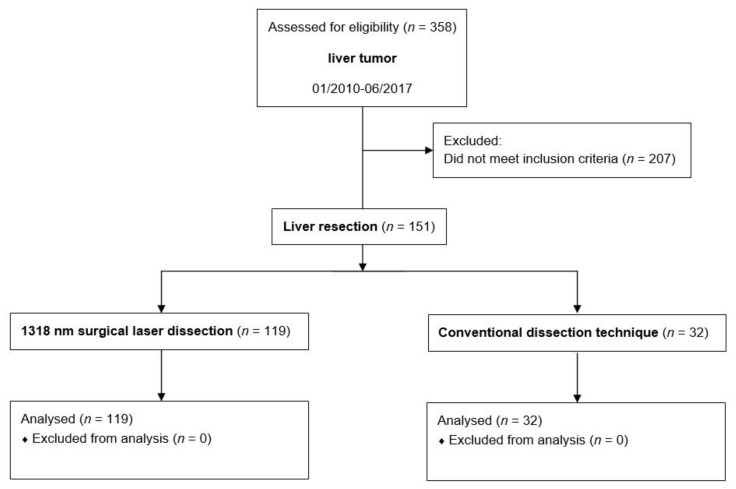
Study flow diagram.

Three hundred and fifty-eight consecutive patients who underwent liver resection between January 2010 and June 2017 were identified from the database regardless of comorbidity, liver disease, structural liver disease or previous oncological therapy. Patients with synchronous procedures, such as cholecystectomies, bile duct resections or biliodigestive anastomoses, which regularly take place as part of liver resections, were included in the analysis. Whenever liver resections were performed synchronously as part of other operations such as HIPEC, multivisceral resections, oncological resections of other organs or as a biopsy, the patients were excluded. Finally, 151 of the 358 patients met the inclusion criteria and could be further analyzed. A flow chart of the study population is shown in Figure 1.

### 2.4. Surgery

Surgery was carried out by dedicated hepatic surgeons with more than five years of experience. To achieve R0-resection, patients received either atypical or anatomical resection depending on the anatomic location of the tumor and previous hepatic operations. Portal triad clamping., i.e., a Pringle maneuver, was not regularly performed. Topical sealants were used at the discretion of the surgeon.

Patients were operated on using general anesthesia supported by a peridural catheter (Th8). Normothermia and a low central venous pressure (0–5 mmHg) were aimed for whenever possible. Except for the dissection procedure, the surgical, perioperative, anesthesiological and intensive care treatment did not vary between patients undergoing laser or conventional dissection.

In patients undergoing laser resection, a 1318 nm Nd-YAG (neodymium-yttrium aluminum garnet) surgical laser that emits laser radiation at a wavelength of 1318 nm (Eraser 1318 nm, Rolle + Rolle GmbH & Co KG, Salzburg, Austria, CE 0123 CB 60601-1) was used. The laser was operated in continuous mode with a power of 60 watts. The laser beam is guided over a handpiece by means of a 3 m-long, flexible quartz fiber light guide with a diameter of 600 µm. A pilot laser projects the application area onto the organ surface. While atypical and circumscribed anatomical liver resections (e.g., segmentectomy, left lateral sectorectomies) were performed without the use of linear staplers, more extensive anatomical and extensive atypical liver resections were carried out with the additive closure of duct structures using conventional surgical techniques such as ties, stitches, clips or power-assisted devices. Central portal vein pedicles or central hepatic veins were divided using linear stapling devices (Endo GIA™ Universal Stapler 45 mm length, loading units 2.5 mm staples, Covidien, Mansfield, MA, USA).

In conventionally resected patients, stapling devices, high frequency instruments or the crush-clamp method were used for dissection.

### 2.5. Patient Characteristics, Parameter of Operative Quality and Outcome Quality, Risk of Bias

Demographic and clinical characteristics including age, sex, and preoperative status according to the American Society of Anesthesiologists (ASA) classification [20,21], diagnosis, type and extent of hepatic resection and histopathological results were analyzed.

The following intraoperative parameters were assessed: operation time, number of stapling devices and sealants used, use and duration of Pringle maneuver, intraoperative blood loss and transfusion. Outcome quality was measured with the following parameters: morbidity, mortality, reoperation and reintervention rate, and length of stay (ICU, hospital), resection margin status [22]. Morbidity was assessed according to the Clavien–Dindo classification of postoperative complications [23,24,25]. Postoperative mortality was recorded and presented as early postoperative (30 day).

### 2.6. Statistical Analysis

Outcomes were compared between the laser and conventional group. Nominally and ordinally scaled variables were presented as absolute and relative frequencies with minima and maxima. Categorical variables were compared between groups using the chi-square test or, when sample size was smaller than five, Fisher’s exact test. For normally distributed variables, means and standard deviations (s.d.) were computed and the distribution was compared using the Student’s *t*-test. For not normally distributed variables, the median and interquartile range (i.q.r) were calculated and they were compared using the Mann–Whitney *U*-test. Test results were considered statistically significant if *p* ≤ 0.050. Statistical analyses were performed using the SPSS^®^ Statistics 27 analysis software (SPSS Inc., Chicago, IL, USA).

## 3. Results

### 3.1. Baseline Characteristics of the Study Population

A total of 151 consecutive patients underwent liver resection during the study period and were included in the analysis. The laser group comprised 119 patients and the conventional group 32 patients. The clinical and demographic characteristics of patients in both groups were comparable (Table 1).

Mean patient age was not different between both groups and there were more males than females in both groups. No statistically significant differences in ASA categorization were observed between groups. The proportion of fibrosis, cirrhosis and fatty liver disease did not differ between groups and neither did the frequencies of underlying pathologies leading to the operation. The extent of hepatic resection did not differ significantly between groups regarding the categories atypical (31.9% vs. 34.4%), minor (42.9% vs. 34.4%) and major (25.2% vs. 31.3%) liver resection.

### 3.2. Parameters of Operative Quality

Table 2 shows intraoperative parameters.

The total duration of the operation did not differ between groups. In the laser group, the number of stapling devices and the frequency of sealants use was significantly lower compared to the conventional group. The need for portal triad clamping (Pringle maneuver) occurred significantly less frequently in the laser group. Median intraoperative blood loss was 200 mL lower in the laser group, and so was the percentage of intraoperatively transfused patients and the amount of transfused PRBCs.

### 3.3. Outcome Parameters

Table 3 summarizes the results regarding outcome quality.

As a marker of oncological safety, the status of the resection margin [22] of patients with malignant disease was compared. There was no difference regarding the proportion of negative margins (R0) as well as the proportion of microscopically positive margins (R1) between the groups. While the length of hospital stay was comparable between the groups, the length of ICU stay was shorter in the laser group. The reintervention rate was significantly lower in the laser group. No difference could be observed between the groups in postoperative 30-day mortality, which was 1.7% in the laser group compared to 3.1% in the conventional group. The morbidity rate was not significantly different between groups and increased in both groups with growing extent of the operation. There was a tendency towards lower grades of complications for the laser group without proof of significance.

## 4. Discussion

The results of this study support the safe feasibility of laser liver resection. It found no statistically significant difference in mortality and morbidity after laser resection compared to conventional resection. The severity of postoperative complications according to the Clavien–Dindo grading system did not differ significantly between the groups. Regarding oncological safety, there was no difference in the frequency of a negative resection margin (R0) between the groups, while intraoperative blood loss, the transfusion of packed red blood cells (PRBCs), the use of Pringle maneuver and the use of sealants were reduced in the laser group.

Mortality is a strong and indispensable quality indicator of any treatment, particularly so in surgery. In line with our results, representative, unselected collectives nowadays show comparable postoperative mortality rates of below 5%. This decrease is attributed to enhanced perioperative care, increasing surgical experience and improved surgical techniques over the past decades [6,8,10,11,12,15]. Against this background, the laser liver resections carried out in our study population appear comparably safe with a mortality of 1.7%, although comparisons across studies have only limited validity given heterogeneity among populations and other potential bias [14,26].

Notwithstanding the decreased mortality, morbidity following liver resection remains high at 35–45% [8,10,11,12,13,14,15,27,28], without noticeable advantage of a single dissection device [9,16,17,26]. Using the Clavien–Dindo Classification, a lower postoperative complication rate was found for laser resections compared to conventional resections. However, given the relatively small number of patients who underwent conventional resection, the power of the analysis was low, and no statistical significance could be reached. A positive association of postoperative complications with the extent of resection was observed for both groups, which is in line with existing evidence [8,11,15,26]. This supports the validity of our morbidity assessment, even though the analysis was retrospective. The proportion of grade III and IV complications was lower in the laser than the conventional resection group. This is of particular relevance because there is evidence suggesting an association between severe, but not mild, postoperative complications and worse oncological outcomes [29,30]. Even though no statistical significance could be shown for the reduced frequency and severity of complications after laser resection, secondary outcomes such as a significantly reduced need for reintervention and reduced length of intensive care unit stay provide indirect evidence of a lower complication severity after laser application. Reinterventions are likely to be associated with longer intensive care unit stay. The available data do not allow for a causal explanation of the lower reintervention rate and shorter intensive care unit stay. Hypothetically, the characteristics and tissue of the dissection technique might influence the risk of complications such as bilioma requiring reintervention.

With the aim of avoiding intraoperative blood loss and transfusions as well as consecutively reducing morbidity and mortality, the use of the Pringle maneuver gained wide acceptance [31,32,33,34]. However, the technique is associated with hepatic ischemia and possible reperfusion damage [35]. Moreover, the spread of tumor cells through post-ischemic reperfusion seems possible based on observations in murine models [36,37]. In the study population, the Pringle maneuver was only used when deemed necessary by the surgeon. In the laser group it was applied significantly less often. In comparable collectives in which other dissection techniques were employed, the frequency with which the maneuver was used was higher [11,14,38].

The intraoperative blood loss in our patients with laser resection was low both in comparison with patients undergoing conventional resection in our study and with patients from other studies where different dissection methods were used [8,10,14,38,39]. A possible explanation for reduced blood loss after laser application is the thermocoagulatory effect of the laser with the formation of a carbonization and necrosis zone of around 2–3 mm in the surrounding tissue. As a result, blood vessels and bile ducts up to 2 mm diameter are securely closed [40]. In analogy to the reduced intraoperative blood loss, transfusions requirements for laser resection were in the lower range of data from other studies [11,14,39]. Reducing blood loss is important for patients because intraoperative blood loss and perioperative transfusions are predictors of postoperative morbidity, mortality and poor long-term results in liver resections [8,10,11,41,42,43,44].

Regarding oncological safety, laser resections show a high rate of microscopically complete resection, which was comparable to patients operated on with alternative dissection techniques in other studies [6,14]. Even in metastatic disease, microscopically complete tumor clearance is crucial and associated with a survival benefit in most settings [45].

There are some methodological limitations to our study. Its retrospective single-center design may limit the external validity of the data. The overall sample size is considerably smaller than the size of for example register studies [6,8,10,11,15], and the conventional resection group is particularly small. The choice of dissection technique was made deliberately by the two individual surgeons and not by randomization, rendering them rather subjective. This makes the study design and the results prone to selection bias. However, patients’ characteristics showed no significant difference between the study groups in terms of age, gender, ASA physical Status, comorbidity, liver parenchyma structure, underlying diagnosis and extent of resection. Long-term survival outcomes are not available so that only surrogate markers for oncological efficacy of the resections could be evaluated. A strength of the study is the consistent use of the standardized and validated Clavien–Dindo Classification to rate postoperative complications [23,24,25]. During the study period, all patients were operated by the same two surgeons or under their direct supervision. Therefore, surgical performance bias, which is a well-known problem [46], should be minimized. The study represents the results of a single tertiary center, and all consecutive patients undergoing liver resection were included in the analysis, thus reflecting clinical reality rather than a highly selected patient group. Future studies, ideally with a randomized controlled design, could provide further insight into the perioperative morbidity and the longer-term results of laser application in liver resections.

## 5. Conclusions

To the best of our knowledge, the present study is the first to describe and demonstrate the safe applicability of laser resection in open liver surgery. Based on its results, these operations can be performed routinely with an acceptable risk for liver resections of any size. Compared to alternative dissection methods, laser resection appears equally safe with comparatively low blood loss and an adequate oncological surrogate outcome.

## Figures and Tables

**Table 1:** Baseline characteristics of the study population.

Patient Characteristic	Laser % (*n* = 119)	Conventional % (*n* = 32)	*p*-Value
Age (years) *	65.4 ± 11.5	64.7 ± 12.8	0.743 (*t*)
Sex			0.269 (*χ*^2^)
Male	58.0 (69)	68.8 (22)	
Female	42.0 (50)	31.3 (10)	
ASA physical status grade			0.146 (*χ*^2^)
1	1.7 (2)	0.0 (0)	
2	30.3 (36)	18.8 (6)	
3	63.9 (76)	68.8 (22)	
4	4.2 (5)	9.4 (3)	
5	0.0 (0)	3.1 (1)	
Parenchymal liver state			0.270 (*χ*^2^)
Fibrosis	10.9 (13)	3.1 (1)	
Cirrhosis	10.1 (12)	12.5 (4)	
Steatosis	5.0 (6)	12.5 (4)	
Normal	73.9 (88)	71.9 (23)	
Diagnosis			0.346 (*χ*^2^)
Primary liver malignancy	19.3 (23)	31.3 (10)	
Secondary liver malignancy	67.2 (80)	56.3 (18)	
Benign liver disease	13.4 (16)	12.5 (4)	
Resected Nodules *			
Number	2.1 ± 2.0	2.1 ± 1.5	0.913 (*t*)
Extend of hepatic resection			0.659 (*χ*^2^)
Atypical	31.9 (38)	34.4 (11)	
Minor	42.9 (51)	34.4 (11)	
Major	25.2 (30)	31.3 (10)	

Values are percentages, values in parentheses are absolute unless indicated otherwise, * values are mean (±s.d.); *χ*^2^ Pearson’s Chi-squared test; *t* Student’s *t*-test.

**Table 2:** Intraoperative parameters.

Operative Parameter	Laser % (*n* = 119)	Conventional % (*n* = 32)	*p*-Value
Duration of operation (min) *	250.2 ± 108.5	243.9 ± 109.3	0.770 (*t*)
Stapling loads (amount) *	2.7 ± 3.6	4.6 ± 5.0	0.017 (*t*)
Sealants			
Use of sealants	37.8 (45)	65.6 (21)	0.005 (*χ*^2^)
Sealants (amount) *	0.5 ± 0.7	0.8 ± 0.8	0.010 (*t*)
Pringle maneuver			
Portal triade clamping	3.4 (4)	15.6 (5)	0.021 (*F*)
Duration of clamping (min) *	7.5 ± 6.4	20.4 ± 9.3	0.051 (*t*)
Total intraoperative blood loss (mL) **	300 (200–550)	500 (212.5–1950)	0.005 (*M*)
Intraoperative transfusion			
Percentage	13.4 (16)	40.6 (13)	0.001 (*χ*^2^)
PRBCs transfused per operation (amount) *	0.4 ± 1.1	2.6 ± 4.9	<0.001 (*t*)

Values are percentages, values in parentheses are absolute unless indicated otherwise, * values are mean (±s.d.), ** values are medians (i.q.r); *χ*^2^ Pearson’s Chi-squared test; *t* Student’s *t*-test; *F* Fischer’s exact test; *M* Mann–Whitney *U* test. PRBC packed red blood cells.

**Table 3:** Outcome quality parameters.

Outcome	Laser % (*n* = 119)	Conventional % (*n* = 32)	*p*-Value
Resection margin			
Negative (R0)	93.2 (96)	89.3 (25)	0.256 (*χ*^2^)
Positive (R1)	5.8 (6)	7.1 (2)	0.077 (*F*)
Length of stay			
Intense care unit (days) **	1 (0–2)	3 (1–5.75)	<0.001 (*M*)
Hospital (days) **	13 (10–19)	14 (9.25–19.75)	0.656 (*M*)
Reoperation rate	2.5 (3)	3.1 (1)	1.000 (*F*)
Reintervention rate	9.2 (11)	21.9 (7)	0.050 (*χ*^2^)
Mortality	1.7 (2)	3.1 (1)	0.513 (*F*)
Morbidity	41.2% (49)	59.4% (19)	0.066 (*χ*^2^)
Postoperative complications (Clavien–Dindo)			0.066 (*χ*^2^)
Grade I	13.4% (16)	15.6% (5)	
Grade II	12.6% (15)	15.6% (5)	
Grade III a	9.2% (11)	15.6% (5)	
Grade III b	2.5% (3)	0.0% (0)	
Grade IV a	1.7% (2)	9.4% (3)	
Grade IV b	0.0% (0)	0.0% (0)	
Grade V (death)	1.7% (2)	3.1% (1)	0.513 (*F*)
Postoperative complications dependent on extent of resection			
Atypical	21.1 (8)	45.5 (5)	0.106 (*χ*^2^)
Minor	43.1 (22)	45.5 (5)	1.000 (*F*)
Major	63.3 (19)	90.0 (9)	0.231 (*F*)

Values are percentages, values in parentheses are absolute unless indicated otherwise, ** values are medians (i.q.r); *χ*^2^ Pearson’s Chi-squared test; *t* Student’s *t*-test; *F* Fischer’s exact test; *M* Mann–Whitney *U* test.

## Data Availability

The data presented in this study are available in article.
